# Structural Analysis of ABC-family Periplasmic Zinc Binding Protein Provides New Insights Into Mechanism of Ligand Uptake and Release

**DOI:** 10.1016/j.jmb.2007.01.041

**Published:** 2007-04-06

**Authors:** Beeram Ravi Chandra, M. Yogavel, Amit Sharma

**Affiliations:** Structural and Computational Biology Group, International Centre for Genetic Engineering and Biotechnology (ICGEB), New Delhi, India

**Keywords:** ABC-type, ATP-binding cassette, PLBPs, periplasmic ligand binding proteins, r.m.s.d, root-mean-square deviation, ZnuA-*Ec*, zinc transporter ZnuA from *Escherichia coli*, ZnuA-*Syn*, zinc transporter ZnuA from *Synechocystis* strain 6803, metal transporter, zinc, crystal structure, ion pairs, domain slippage

## Abstract

ATP-binding cassette superfamily of periplasmic metal transporters are known to be vital for maintaining ion homeostasis in several pathogenic and non-pathogenic bacteria. We have determined crystal structure of the high-affinity zinc transporter ZnuA from *Escherichia coli* at 1.8 Å resolution. This structure represents the first native (non-recombinant) protein structure of a periplasmic metal binding protein. ZnuA reveals numerous conformational features, which occur either in Zn^2+^ or in Mn^2+^ transporters, and presents a unique conformational state. A comprehensive comparison of ZnuA with other periplasmic ligand binding protein structures suggests vital mechanistic differences between bound and release states of metal transporters. The key new attributes in ZnuA include a C-domain disulfide bond, an extra α-helix proximal to the highly charged metal chelating mobile loop region, alternate conformations of secondary shell stabilizing residues at the metal binding site, and domain movements potentially controlled by salt bridges. Based on in-depth structural analyses of five metal binding transporters, we present here a mechanistic model termed as “partial domain slippage” for binding and release of Zn^2+^.

## Introduction

**Figure 1:**
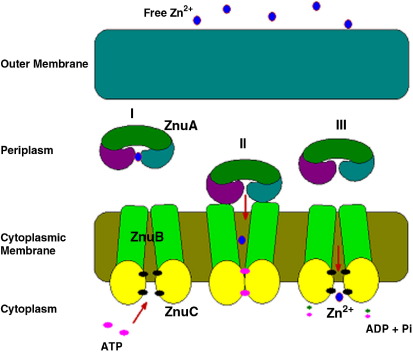
A model for Zn^2+^ transport in bacterial systems. The three stages are as follows: (I) ZnuA binds Zn^2+^, (II) ZnuA and ZnuB bind each other and ZnuA transfers Zn^2+^ to ZnuB, and completion of the Zn^2+^ transport cycle (III).

The ATP-binding cassette (ABC-type) superfamily of transport systems consists of several protein complexes which together are capable of transporting various size solutes across membranes^1^^,^^2^ (e.g. Zn^2+^ transporter; Figure 1). The periplasmic ligand binding proteins (PLBPs) recognize diverse ligands and deliver their cargo to membrane-bound members of the ABC-type family for selective uptake into the cytoplasm. Metal binding PLBPs show limited sequence homology but structurally retain the overall bi-lobed, pseudo-symmetric structures where N and C-terminal domains are linked by either flexible β-strands^3^ or *via* a long linker helix.^1^

ABC-type PLBPs from a number of bacteria have been classified into clusters on the basis of sequence homology and ligand identities.^4^ The newly defined cluster 9 family comprises of zinc transporter ZnuA from *Escherichia coli* (ZnuA-*Ec*)^5^ and *Synechocystis* strain 6803 (ZnuA-*Syn*),^6^ the manganese transporter PsaA from *Streptococcus pneumoniae*^7^ and the zinc transporter TroA from *Treponema palladium*.^8^ PsaA and TroA have been proposed to transport both zinc and manganese ions.^9^ Crystal structures have also been reported for several large size ligand binding PLBPs. Data from the widely studied maltose binding PLBP suggested that during ligand engagement the N and C-terminal domains undergo ligand dependent conformational change and rotate ∼35° about a hinge region.^10^ Most other large ligand PLBPs have similar molecular architectures to maltose binding PLBPs, and presumably function using a similar ligand-induced “Venus fly-trap” or “clamping” mechanism. However, structures of metal binding PLBPs of cluster 9 family display a distinct fold wherein a rigid α-helix connects the two terminal domains. Rigid nature of this connecting α–helix suggests that this subclass of PLBPs do not undergo large scale conformational changes as in type I and II PLBPs. This suggestion is validated by comparison of free^10^ and metal bound^8^ TroA structures, where the release of Zn^2+^ results in an ∼1 Å net movement of the two domains toward one another without any obvious large-scale conformational changes in peripheral regions.

A comprehensive structural analysis of metal binding PLBPs (ZnuA-*Syn*, PsaA, TroA and MntC)^6–8,11,12^ reveals variable solute binding pockets. Though the crystal structures of these four metal binding PLBPs are available at high resolution, a testable model for ion uptake and release remains elusive. More significantly, the metal ion specificity of some of these transporters has been debated. Crystal structures of PsaA^7^ and TroA^8^ revealed zinc in the solute binding pockets while *in vivo* and *in vitro* data indicated that manganese was likely to be the natural ligand.^13^ Recombinant hyper-expression of these proteins for crystal structure analysis is argued to be one possible reason for such discrepancies.^1^ It is proposed that chaperones and peptidyl-prolyl isomerases in recombinant expression hosts (like *E. coli*) differ from those in cognate hosts in their specificity, and therefore may induce partly incorrect folding and allow binding of non-physiological metal ions.^1^^,^^13^ Therefore, a crystal structure of a native (non-recombinant) PLBP with physiologically bound metal ion in its solute binding pocket has been much awaited to understand the mechanism of ion uptake and release by these transporters. Towards this end, we have determined the crystal structure of native (non-recombinant) ZnuA from *E. coli* in a naturally zinc bound state. Our analyses are therefore free both from issues arising from recombinant protein over-production using heterologous expression systems, and questions raised by *in vitro* loading of metals prior to protein crystallization. The presented work therefore serves as an authentic conformational state of a natively Zn^2+^ bound ABC-type PLBP from the cluster 9 family. Comparative analysis of ZnuA-*Ec* with conformational states of other metal bound PLBPs has allowed us to propose a mechanism for ion uptake and release based on small but distinct conformational changes.

## Results and Discussion

### Overall structure of ZnuA-*Ec*

**Figure 2:**
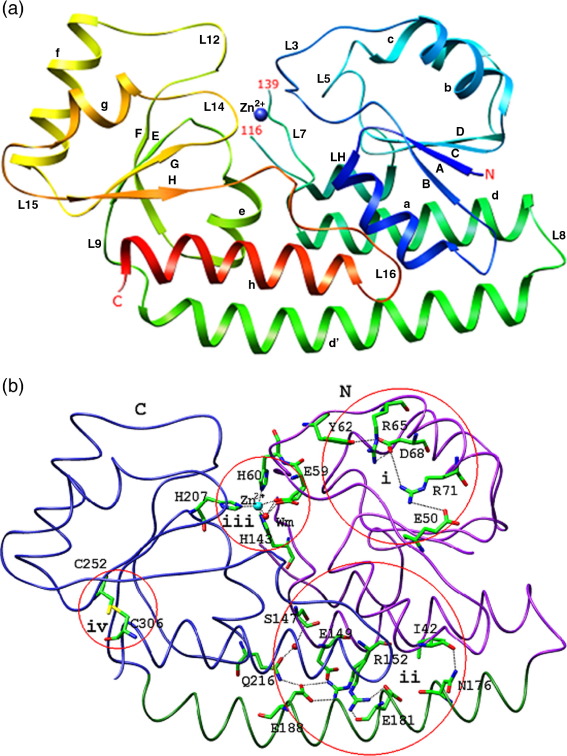
Ribbon diagram of the overall structure of ZnuA from *E. coli*. (a) ZnuA-*Ec* displays a C-clamp like fold with a pseudo-2-fold axis linked by a long helix. N-domain (purple), C-domain (medium blue) and long helix (forest green) are colored differently. Bound Zn^2+^ is represented as an orange sphere and the protein termini are marked. (b) An overall illustration of important features of molecule A of ZnuA-*Ec*. The marked labels are as follows: (i) hydrogen bonding network at the metal binding arm; (ii) domain movement controlling the hydrogen bonding network and salt brides; (iii) metal binding site; (iv) unique disulfide bond in the C-domain region.

The asymmetric unit is composed two protomers of ZnuA (molecules A and B; 520 residues), and includes two zinc ions and 527 water molecules. Superposition of the two independent molecules in the asymmetric unit yields a root-mean-square deviation (r.m.s.d.) value of 0.3 Å for 933 backbone atoms and therefore we are, for the most part, presenting and discussing molecule A. Structure of ZnuA-*Ec* possesses a pair of (α/β)_4_ sandwich domains which are held together by a long linker α helix that is tightly packed against them (Figure 2(a)). Similar to other ABC-type PLBPs of cluster 9 family, ZnuA-*Ec* displays pseudo-2-fold symmetry between the two terminal domains. Numbering of the α-helices, β-strands and loops are denoted as A-H, a-h and L1–L16, respectively. The additional α-helix found in N-domain in the loop L7 region is labeled as LH. The N-terminal domain (N-domain, residues 27–166) and the C-terminal domain (C-domain, residues 200–308) possess 2-3-1-4 linked parallel (α/β)_4_ topologies and superimpose with a r.m.s.d. value of 2.7 Å (for 75 C^α^ atoms) despite having sequence identity of less than 20%. As in other ABC-type PLBP transporters, the metal binding site is located at the interface between two terminal domains (Figure 2(a)). The unique structural features of ZnuA-*Ec* are shown in Figure 2(b) and described below.

### Comparison with other ABC-type PLBPs

**Supplementary Figure SF4:**
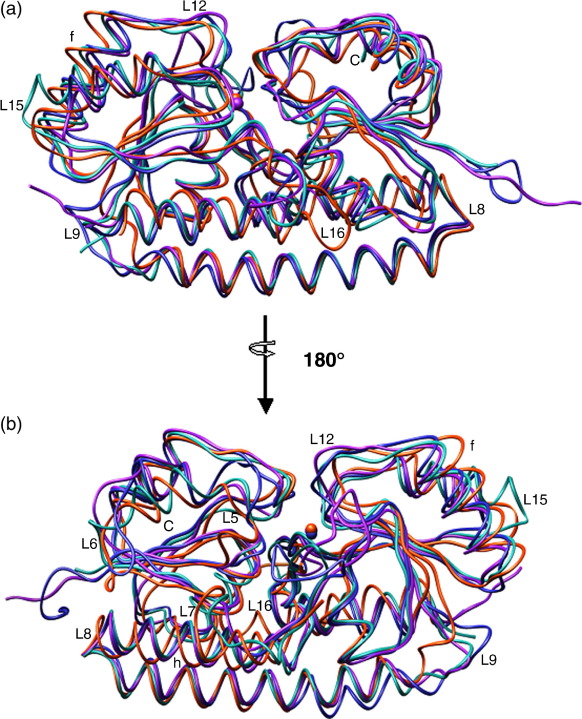
Two views of superimposed line representations of ZnuA-*Ec* along with two other metal binding PLBPs showing the highly conserved folds. The largest conformational differences occur in the C-domain loop region, which is proximal to interface between C-domain, N-domain and the long helix. ZnuA-*Ec* (red orange) TroA (light sea green), PsaA (medium blue) and MntC (purple) are superimposed along with their metal components.

**Figure 3:**
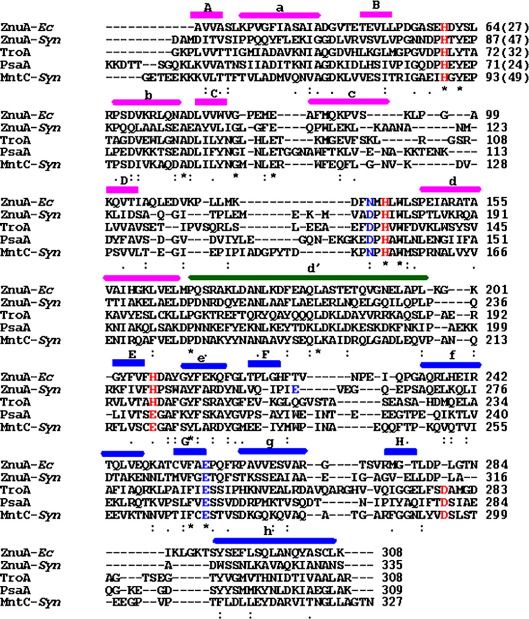
Structure-based sequence alignment of metal binding PLBPs from the ABC-type cluster 9 family. Proteins are: ZnuA-*Ec* (*Escherichia coli*), ZnuA-*Syn* (*Synechocystis* 6803), TroA (*Treponema pallidum*), PsaA (*Streptococcus pneumoniae*) and MntC-*Syn* (*Synechocystis* 6803). Metal binding residues are highlighted in red and their secondary shell stabilization residues are highlighted in blue. Twelve identical/strictly conserved residues, 37 conserved residues and 24 semi-conserved substitutions are marked with an asterisk, semicolon and dot, respectively.

**Figure 4:**
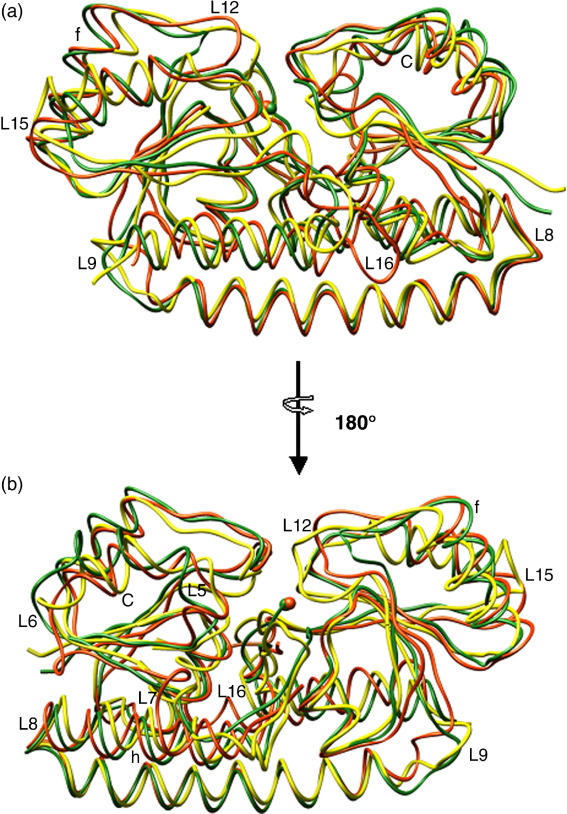
Two views of superimposed line representations of ZnuA-*Ec* along with ZnuA-*Syn* and metal unbound TroA showing the highly conserved folds. The largest conformational differences occur in the C-domain loop region, which is proximal to the interface between the C-domain, N-domain and the long helix. ZnuA-*Ec*, ZnuA-*Syn* and metal unbound TroA are shown in red, orange forest green, and yellow, respectively. The bound Zn^2+^ is shown in sphere representation.

Even though sequence identity of ZnuA-*Ec* with other ABC-type metal binding PLBP transporters is less than 30%, structural congruence is very high (Figure 3). The structure of ZnuA-*Ec* was superimposed with other PLBPs and overlap of 229, 234, 233, 231 and 231 C^α^ atoms yielded a r.m.s.d. value of 1.7 Å (ZnuA-*Syn*),^6^ 1.9 Å (TroA),^8^ 1.9 Å (metal free TroA),^9^ 1.9 Å (PsaA)^7^ and 1.8 Å (MntC),^12^ respectively (Figure 4(a) and Supplementary Data, Figure SF4a). However, it is clear that there are several regions of conformational differences. Close to the N-domain, a large conformational difference is observed in the region of helix c and loop L5. Interestingly, an additional helix LH is found in the N-domain of ZnuA-*Ec* (Figure 1). Further, the charged loop L7 (residues 144–148) has moved ∼2–3 Å away from the bottom of the metal binding cleft and this reflects slight movement of helix d in the N-domain as well as a ∼4 Å movement of helix h in the C-domain. These structural movements may be due to closed conformation of loop L16 (residues 282–291). Entrance of the metal binding cleft (loop L12) in ZnuA-*Ec* and ZnuA-*Syn*^6^ is different from those in PsaA,^7^ TroA^8^ and MntC^12^ structures. In the former two, loop L12 has moved ∼4 Å away from the cleft and therefore makes the metal binding site more accessible to solvent. Intriguingly, there are large deviations observed in loop L16 (residues 282–291) in the C-domain, and this loop adopts a closed conformation in ZnuA-*Ec* in comparison to the other four structures (Figure 4(b) and Supplementary Data, Figure SF4b).

### Zinc in the metal binding pocket

We propose that the ion bound in the metal binding pocket of native ZnuA-*Ec* is Zn^2+^. This metal comes from *E. coli* as Zn^2+^ was not a component of buffers used during purification and crystallization steps. Selenium- single wavelength anomalous dispersion data were collected at a wavelength of 0.974 Å (energy = 12.730 keV) for structure solution. At this wavelength, the *f*ʺ values for Zn^2+^ and selenium are 3.84 e^−^ and 2.47 e^−^, respectively (compared to 1.30 e^−^ for Mn^2+^). Indeed, substructure solution gave Zn^2+^ as the top two peaks (confirmed later by anomalous difference maps). Therefore, ZnuA-*Ec* represents the first high-resolution structure of a native, non-recombinant metal transporter.

**Figure 5:**
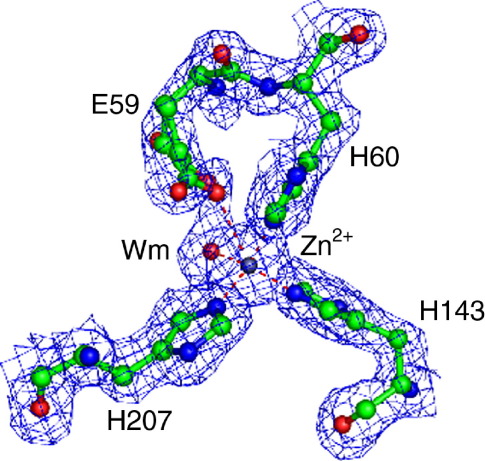
Metal binding residues of ZnuA-*Ec*. The overlayed electron density map (2*F*_o_-*F*_c_) is contoured at the 1.0σ level. Bound zinc is held by His60, His143, His207 and a water molecule, which is found at the apex coordination site. Two of the three metal binding residues are contributed by the N-domain and His207 comes from the C-domain. In addition, Glu59, which has a double conformation, makes a long coordination bond with zinc.

The metal binding pocket in ZnuA-*Ec* is formed at the interface of the N and C-domains. Zinc is coordinated by residues His60, His143, His207 and a water molecule (Figure 5). Well-defined and continuous electron density is observed for all the active site residues, which make tetravalent interaction with the bound zinc. While the histidine residues coordinate zinc v*ia* N^ε2^ atoms in a trigonal pyramid geometry, the fourth ligand water occupies the apical position. The metal bound water has *B*-factors of 10.6 Å^2^ and 6.2 Å^2^ for molecules A and B, respectively. Interestingly, Glu59, which is close to the active site water, makes a direct long coordination bond with Zn^2+^ (2.8 Å in molecule A and 3.3 Å in molecule B). This difference is likely to have implications with respect to metal bound and unbound states of ZnuA-*Ec*.

In the case of ZnuA-*Ec* and ZnuA-*Syn*,^6^ the C-terminal domains contribute only one residue (histidine) for zinc coordination. However, in case of PsaA,^7^ TroA^8^ and MntC^12^ structures the metal binding pocket is formed by two residues from each of the N and C-domains. Based on sequence comparisons, Asp279 in ZnuA-*Ec* and Asp313 in ZnuA-*Syn*^6^ are conserved amongst ABC-type PLBPs and these are expected to be the fourth critical residue in the metal binding pocket as observed in the case of PsaA and TroA structures. However, Asp279/Asp313 do not make any contribution to the metal binding in the case of ZnuA-*Ec* and ZnuA-*Syn*. Further, unlike in ZnuA-*Syn*, TroA and PsaA structures, neither ZnuA-*Ec* nor MntC contain the tripeptide DPH motif near active site residues His60 and His143. These differences among the metal binding PLBPs clearly highlight divergence within the cluster 9 family.

### Unique disulfide bond

The cynobacterial manganese transporters have conserved cysteine residues which form a disulfide bond.^12^^,^^14^ Structural analysis of MntC from *Synechocystis* reveals a Cys219–Cys268 disulfide bond located between strands E and G.^12^ This S–S bridge is missing in ZnuA-*Ec*, ZnuA-*Syn*, TroA and PsaA and its significance remains unclear. Intriguingly, however, ZnuA-*Ec* possesses a unique disulfide bond (Cys252–Cys306) in an unexpected location, between strand G and helix h (Figure 2(b)). This disulfide bond is surface exposed, and is a novel feature of ZnuA-*Ec*, as it is absent from homologous zinc transporters. The accessible nature of this S–S link in ZnuA-*Ec* lends it amenable to reduction in the periplasmic environment but its significance remains unclear.

### The β-strands architecture

**Figure 6:**
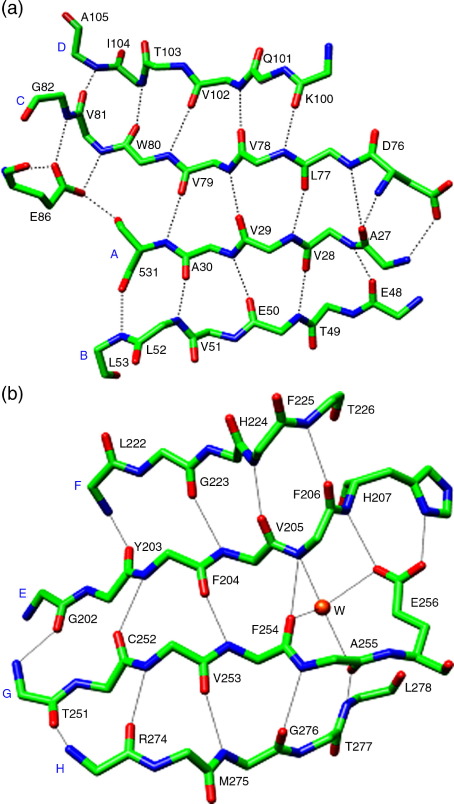

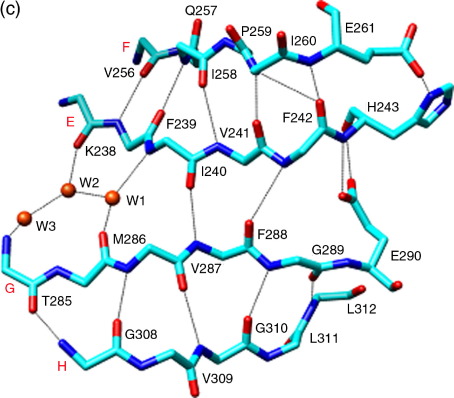
β-Strand architecture of N and C-domains. (a) Metal binding PLBPs have very similar backbone hydrogen bonding arrangements in their N-domains. Positions 86 and 82 are strictly conserved for Glu and Gly, respectively, while positions 31 and 79 are conserved for Ser/Thr and Asp/Glu, respectively. (b) Four backbone hydrogen bonds and water-mediated hydrogen bonds are present between strands E and G of ZnuA-*Ec*. Side-chain atoms of conserved Glu256 hydrogen bond to backbone N and side-chain N^δ1^ of a metal binding residue. (c) A different architecture of the β-strands in C-domains of ZnuA-*Syn*, TroA, PsaA and MntC structures. In the latter group, a conserved water molecule (this is corresponding to the one involving backbone N and O atoms at positions 286 and 240) is present. In ZnuA-*Syn*, His243 is flipped 180° compared to ZnuA-*Ec* and TroA.

The association of β-strands in C-terminal domain of ZnuA-*Ec* (Figure 6) is different when compared to ZnuA-*Syn*, TroA, PsaA and MntC structures. There is a large separation found at the C-terminal end of strands E and G in ZnuA-*Ec*. Interestingly, in the case of ZnuA-*Syn*, the gap between E and G strands is bridged by three bound water molecules (Figure 6(c)), of which one water molecule is conserved in all three structures PsaA,^7^ TroA^8^ and MntC.^12^ These features suggest that water is likely to play key roles during the small conformational changes that occur during metal coordination and release. Further, Glu256 (strand G) is conserved in all the five proteins (TroA, PsaA, MntC, ZnuA-*Syn* and ZnuA-*Ec*) and this residue is involved in side-chain mediated interactions with backbone N atoms of strand E (in which one of the residues is the metal chelating His/Glu). In addition, one of the side-chain oxygen atoms of Glu256/248 hydrogen bonds to sidechain N^δ1^ of metal binding His207/199 in the case of ZnuA-*Ec* and TroA. However, the corresponding active site His243 in ZnuA-*Syn* is flipped 180° in a trapdoor-like fashion compared to ZnuA-*Ec* and TroA structures. This histidine flipping is likely to correspond to metal bound and release states of metal transporting PLBPs, as discussed below.

### Salt bridges and conserved hydrogen bonding networks

**Supplementary Figure SF7:**
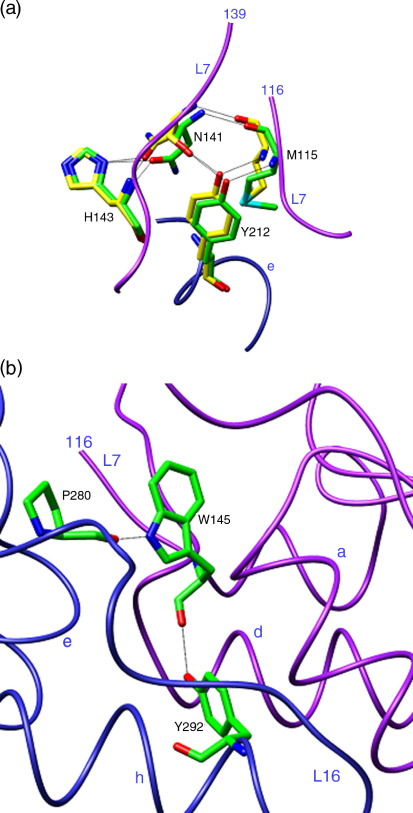
(c) The conserved hydrogen bonding network at loop L7 (d) The conserved hydrogen bonding network at bottom of the metal binding cleft which links tightly for bottom part of the N- and C-domains.

**Figure 7:**
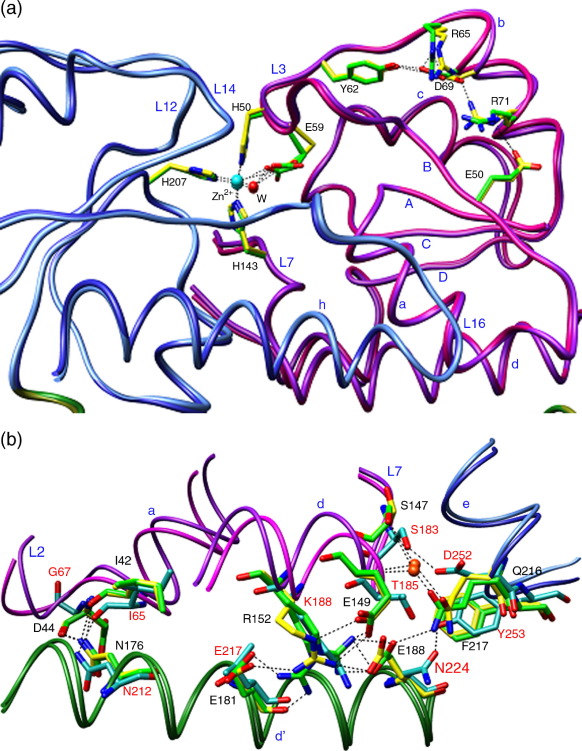
Hydrogen bonding network and inter-domain interactions in ZnuA structures. (a) Superposition of A and B monomers of ZnuA-*Ec* from the asymmetric unit is shown. The N-terminal metal binding arm of ZnuA has strictly conserved Tyr62 and Asp68 which hydrogen bond. The salt bridge networks between Arg65, Arg71, Glu50 and Asp68 are also shown. These interactions may restrict the movement of the metal binding arm, which holds residues His60, and Glu59. (b) The conserved hydrogen bonding networks and unique salt bridges for ZnuA-*Ec* and ZnuA-*Syn* are shown. A and B versions of ZnuA-*Ec* are shown in green and yellow whereas ZnuA*-Syn* is shown in sea blue.

ZnuA-*Ec* structure reveals two important salt bridges occurring in the N-domain metal binding arm (which comprises of strand B, loop L3 and helix b) and the long linker helix d'. In the metal binding arm region, a conserved hydrogen bond is observed between side-chains Tyr-Asp/Gln for ABC-type PLBPs of cluster 9 family structures. The interaction distances range from 2.5 Å to 3.2 Å, except in molecule B of ZnuA-*Ec* (3.9 Å) and PsaA (4.1 Å). These differences are significant, and most likely provide snapshots of transitional states during metal binding and release. Superimposition of molecules A and B of ZnuA-*Ec* reveals small but significant deviations near the helix b region. In the metal binding arm of ZnuA-*Ec*, there is a salt bridge network (between Arg65, Arg71, Asp50 and Asp68), which extends to form the metal binding pocket (Figure 7(a)). These salt bridge linkages restrict the movement of residues Glu59 and His60 and lock the active site amino acid cluster in a defined state. The metal binding residue His143 is located at the base of mobile loop L7. This residue is also stabilized by a secondary shell hydrogen bonding network (Supplementary Data, Figure SF7c). The termini of loop L7 residues are close to each other and are linked by backbone hydrogen bonds between Met115 and Asn141. These interactions are further reinforced by the strictly conserved residue Tyr212 side-chain hydrogen bond. Asn141 is a secondary shell stabilizing residue and in close interaction with active site His143. Interestingly, in A and B representations of ZnuA-*Ec*, the side-chain of Asn141 adopts different conformations. This residue position is conserved in all the cases of ABC-type metal binding PLBPs and occurs either as Asn or as Asp.

At the bottom of the metal binding cleft, the N-domain Trp145 is strictly conserved amongst most metal binding PLBPs of cluster 9 family. Trp145 is involved in a hydrogen bonding network with C-domain Pro280 and Tyr292 (Supplementary Data, Figure SF7d). The conservation of this hydrogen bond network may hold the bottom portion of the N and C-domain tightly upon binding and release stages. In addition, in middle of the long helix d', residue Leu184 is strictly conserved in all metal binding PLBPs, suggesting that this hydrophobic lock may restrict movement of the N- domain. A salt bridge is found between N-domain residues Arg152 and Glu149 and the helix d' residues Glu181, and Glu188 (Figure 7(b)). In molecule A, Arg152 adopts a double conformation suggesting flexibility. This salt bridge extends to form a hydrogen bond network with C-domain residues. The side-chain oxygen atom of Glu188 forms a hydrogen bond with N^ε2^ of Gln216 (2.6 Å) in molecule A while O^ε1^ is involved in water mediated interactions with Ser147. However, in molecule B, the distance between O^ε2^ of Glu188 and N^ε2^ of Gln216 is large (4.1 Å), while Ser147 again adopts a double conformation. Hence, movement of the C-domain helix e is likely to act like a relay bar in a “seesaw” mechanism of conformational change.

### Conformational differences within ABC-type PLBPs

Crystal structures of metal transporting PLBPs indicate that their N-terminal domains are highly conserved. For example, the backbone hydrogen bonding pattern between N-domain β-strands are very similar to one another for all structures analyzed from this protein family. There are also no large deviations found in the long linker helix in metal binding PLBPs either in metal bound and free forms, indicating rigidity of this linker segment. However, the C-terminal domains seem more flexible in these metal-binding PLBPs. A large separation between β-strands E and G in the C-terminal domain is evident for ZnuA-*Syn*,^6^ TroA^7^ and MtnC^12^ structures. The largest conformational differences in the form of unwinding of helices occur for helices c and f, which are located at the surface of the protein (Figure 4). These structural changes potentially hint at events that may occur upon binding of the PLBPs to their counter parts on the integral membrane protein, ZnuB in this case.

### Model for metal uptake and release

**Supplementary Figure SF8:**
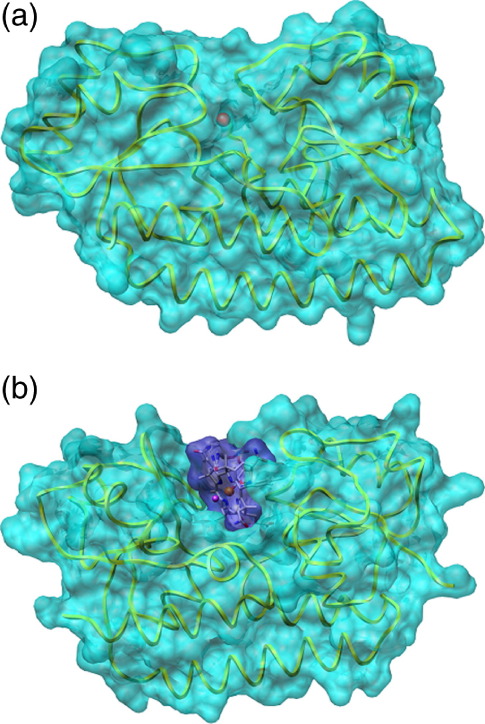
Surface representation of ZnuA-*Ec* and vitamin B12 bound BtuF molecules and the surface is illustrated on 50% transparency. (a) In ZnuA-*Ec,* the bound zinc atom is shown in red sphere. (b) Vitamin B12 is shown in stick with C, O, N and P atoms in gray, red, blue and cyan, respectively. The Co and Cl ions are shown in orange and purple sphere, respectively.

**Figure 8:**
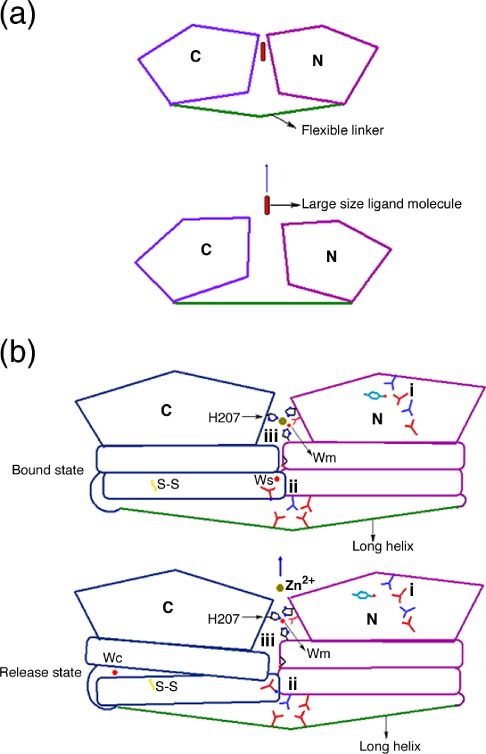
(a) The Venus fly-trap mechanism proposed for capture/release of large ligands by PLBPs. The N and C-domains are held by a flexible linker which acts as a hinge. Bending of the flexible linker allows opening of the two domains so that the trapped ligand can escape. (b) Our proposed partial domain slippage mechanism for capture/release of zinc by cluster 9 family of PLBPs. The conserved hydrogen bonding networks and salt bridges restrict movement of the metal binding arm, and upon metal release a water and Glu59 will occupy the vacant metal site. The alternate conformation of Arg152 and a seesaw mechanism of C-domain helix e may cause the slippage of the bottom part of the C-domain. The breakage of backbone hydrogen bonds between strands G and E in the C-domain may trigger flexibility that leads to flipping of the active site His207, which was seen in the ZnuA*-Syn* (Figure 6(c)). Flipping of His207 may be the first conformational step towards release of bound metal. The marked labels are as follows: (i) hydrogen bonding network at the metal binding arm; (ii) domain movement controlling the hydrogen bonding network and salt brides; (iii) metal binding site. Wm, Wc, Ws are metal bound, conserved and structural water molecules, respectively.

The binding clefts of metal binding PLBPs are narrower and deeper than their counterparts which transport larger size ligands.^15^ The N and C-domain interface in metal binding PLBPs is rather hydrophilic, whereas this region is distinctly hydrophobic for PLBPs which carry ligands like vitamin B12.^16^ The atomic design of PLBPs is therefore both elegant and intuitive – ligand binding pockets are simply covered by longer loops in metal transporting PLBPs (Supplementary Data, Figure SF8a) whereas shorter loops in the large ligand PLBPs allow for a wider and more open interface which larger solutes can use (Supplementary Data, Figure SF8b). It is also becoming clear that the metal binding PLBPs utilize a different mechanism for ligand binding and release than the PLBPs which target larger size ligands (the so-called “Venus fly-trap” mechanism). Our structural analysis suggests that the Venus fly-trap mechanism, which is based on significant motion of the hinge linker between N and C-domains (Figure 8(a)) seems unlikely to be applicable for the metal transporting PLBPs. Instead, we propose a “partial domain slippage” mechanism which stems from small yet significant conformational changes in multiple locations of ZnuA, which together potentially drive binding and release of zinc (Figure 8(b)). The following salient features support our proposal of metal binding and release mechanism termed as partial domain slippage.

(i) The important loop L9 in the C-domain is longer than the corresponding loop L8 in the N-domain, and it adopts variable conformations in metal binding PLBP structures analyzed so far. Therefore, L9 may play a critical role during partial domain collapse. A similar long loop is absent between N-domain and the backbone helix d'. Instead, the N-domain loop L8 adopts nearly identical conformations in all metal binding PLBP structures available. This shorter N-domain loop L8 is therefore likely to control the limited movement within N-domain.

(ii) The salt bridges and conserved hydrogen bonding networks between Tyr62 and Asp68 in the N-domain of ZnuA sufficiently constrain movement of the metal binding arm, which holds key residues Glu59 and His60 in a bound state of ZnuA. During metal release, the metal binding arm may move towards the binding cleft such that a water molecule and Glu59 occupy the vacant metal site (Figure 7(a)). Glu59 adopts a double conformation and is proximal to active site in molecule A but not in B, suggesting structural polymorphisms very close to the active site.

(iii) The two conserved hydrogen bonding networks between (a) Tyr292, Met115 and Asn141 (Supplementary Data, Figure SF7c) and (b) Trp143, Tyr212 and Pro180 (Supplementary Data, Figure SF7d) hold the bottom part of the N and C-domains. This hydrogen bonding network is conserved regardless of whether the metal binding PLBP is in a bound state (ZnuA-*Ec*) or in a potential release state (ZnuA-*Syn*).^6^ This network therefore restricts movement of N and C- domain bottoms thereby making the Venus fly-trap mechanism unlikely for metal binding PLBPs.

(iv) The conserved main-chain (Ile/Val42) and side-chain (Asn/Arg176) hydrogen bonds between N-domain helix a and long helix d', along with the salt bridge between Arg152, Glu49, Glu181, Glu188 together lock the N-domain with the hinge backbone helix d'. This imposes bending restrictions on the backbone helix, and disallows opening of the N-domain. Once again, this conformational restriction makes the Venus fly-trap mechanism for metal binding PLBPs unlikely. Therefore, the “seesaw” mechanism of C-domain helix e detailed earlier may facilitate slippage of the bottom part of the C-domain, which is necessary prerequisite for flipping of His207, which in turn may initiate early steps in metal release.

(v) In ZnuA-*Syn*^6^ (and also in PsaA,^7^ TroA,^8^ and MtnC^12^), which we believe represents metal binding PLBP in a release state, there is a conserved water molecule between C-domain strands E and G (Figure 6(c)). However, in ZnuA-*Ec*, which we propose presents a snapshot of the bound state, there is no equivalent water. Entry of water in the C-domain is likely to break a series of backbone hydrogen bonds between strands E and G (as observed in ZnuA-*Syn*), which in turn may facilitate slippage of the top portion of the C-domain.

(vi) The C-domain metal binding His207 from ZnuA-*Ec* and the equivalent His243 of ZnuA-*Syn* are flipped 180° relative to each other, without any obvious conformational changes in other N-domain metal binding residues. This histidine conformation in ZnuA-*Syn* is likely to correspond to the metal release step. A particular trigger is required to cause conformational movements, which lead to the flipping of His207/His243 (Figure 8(b)). Movement of the metal binding arm towards the metal binding cleft along with breakage of backbone hydrogen bonds between β-strands E and G in the C-domain (the hydrogen bonding network here is again different between ZnuA-*Ec* and ZnuA-*Syn*) may together provide such a trigger.

Therefore, we propose a chain of events that are initiated by binding of loaded ZnuA to ZnuB. Clearly, docking of ZnuB on to ZnuA may provide energy for triggering domain movements and conformational changes, which will eventually lead to the release of bound zinc. Complexing of ZnuA with ZnuB may move the N-domain metal binding arm closer to the zinc binding cleft. Then, water may enter the C-domain sheet regions and lead to slippage of the C-domain top part. This conformational alteration may translate into flipping of the active site His207 and subsequent steps of metal release. The cycle of binding and release will then be completed when the collapsed C-domain moves away from the active site due to import of a new zinc ion. In summary, the structure of ZnuA from a native, non-recombinant source has allowed us to compare and analyze various modes of metal chelation and release within the PLBP cluster 9 family. Our analyses provide evidence for step-wise release of bound zinc from ZnuA which stems from small conformational changes in the C-terminal domain of ZnuA. In this light, we assign the present ZnuA-*Ec* complex with zinc as the metal “bound” state while other previous structures like ZnuA-*Syn* may represent initial conformational steps of the metal “release” state. The series of small structural changes required for egress of metal form part of our proposed partial domain slippage mechanism, which is in sharp contrast to the Venus fly-trap mechanism, which seems unlikely to be applicable to high-affinity metal transporters.

## Materials and Methods

### Protein purification and crystallization

*E. coli* strain B834 (DE3) (derivative of K-12) was grown in M9 minimal medium supplemented with 0.2% (w/v) glucose, 50 μg ml^−1^ of each amino acid including selenomethionine (except Met and Cys) according to the standard selenomethionine labeling protocol.^17^ The culture was centrifuged after 24 h of growth and the cell pellet was resuspended in lysis buffer (25 mM Tris –(pH 8.0), 300 mM NaCl) and lysed by sonication. The supernatant was collected by centrifugation and passed through Ni-NTA column, where native (non-recombinant) ZnuA-*Ec* bound tightly, perhaps due to the presence of histidine and acidic residue stretches in the protein. Bound proteins were washed with lysis buffer and eluted with lysis buffer containing 100 mM imidazole. Eluted fractions were dialyzed against 25 mM Tris –(pH 8.0), 25 mM NaCl and later loaded on to a Q-Sepharose column. Proteins were eluted using a 25 mM to 2 M shallow linear gradient of NaCl in 25 mM Tris –(pH 8.0) buffer. Bound proteins were eluted in various fractions and a specific fraction enriched in ∼35 kDa protein was identified and further purified using gel filtration chromatography on S200 column in buffer containing 50 mM Tris –(pH 8.0) and 300 mM NaCl. A monodisperse peak was collected off the gel filtration column and protein purity verified on SDS–12% (w/v)PAGE. The protein (ZnuA) was concentrated to 5 mg ml^−1^ and buffer exchanged into 25 mM Tris –(pH 8.0) and 25 mM NaCl. ZnuA was crystallized using hanging drop vapor diffusion technique by mixing 1 μl each of protein and mother liquor (20% (w/v) PEG 3350 and 200 mM ammonium sulfate). Crystals appeared in a week and were used for data collection under cryogenic conditions.

### Data collection and structure determination

The crystal chosen for data collection was transferred to the same buffer medium used for its growth, which was supplemented with 20% (v/v) ethylene glycol. Single wavelength anomalous dispersion data were collected at the selenium absorption edge (λ = 0.974 Å) at beamline XRD1, Elettra, Trieste, Italy using one frozen crystal (at 100 K). Diffraction data were reduced and scaled using MOSFLM^18^ and SCALA,^19^ respectively. Eleven of the 12 possible Se and two Zn atoms were found and refined at 2.5 Å resolution using SOLVE.^20^ The mean figure of merit was 0.36 and subsequent processing with RESOLVE^21^ allowed automatic building of 71% of the residues. The partial model was subsequently completed and further refined using REFMAC^22^ to *R*-factor and *R*_free_ values of of 22.5 and 26.7%, respectively. Manual model adjustments were made using Coot.^23^ Model refinement was performed without imposing NCS restraints so that conformations of the two different molecules in the asymmetric unit could be determined independently. The N-terminal 26 residues, a charged loop of 22 residues (116–138) and two residues from the C-terminal are disordered and therefore invisible in the electron density maps. Stereochemistry of the final model was verified using PROCHECK^24^ (Table 1). Structural superimpositions were done using ALIGN.^25^ Structure-based sequence comparison and all figures were prepared using CHIMERA.^26^

### Protein Data Bank accession code

Atomic coordinates for ZnuA-*Ec* have been deposited in The RCSB PDB with accession number 2OGW.

## Figures and Tables

**Table 1:** Statistics of diffraction data and structure refinement

A. *Data collection*	
Space group	*P*2_1_2_1_2_1_
Unit cell dimensions (Å)	*a* = 72.90, *b* = 86.38, *c* = 87.99
Wavelength (Å)	0.974
Resolution range (Å)	61.66–1.82
Unique reflections	49,689 (3441)
Completeness (%)	100 (99)
*I*/σ(*I*)	16.68 (3.99)
*R*_sym_	0.055 (0.25)
Multiplicity	10.6 (8.7)

B. *Refinement*	
Working set	47173
Test set (5.1%)	2516
*R*-factor / *R*_free_ (%)	22.54 / 26.86

C. *Model compositions*	
Amino acids (A and B chains)	520
Total number of protein atoms (partial occupancy)	4171 (318)
Metal atoms	2
Water molecules	527

D. *Stereochemistry*	
Bond lengths (Å)	0.02
Bond angles (°)	1.49
Chiral center restrains (Å^3^)	0.11

E. *Ramachandran plot*	
Residues in most favored regions (%)	97.1
Residues in additional allowed regions (%)	2.9

F. *Mean* B-*factors* (Å^2^)	
All protein atoms	15.6
Main-chain atoms	15.6
Side-chain atoms	17.1
Metal ions	13.5
Water molecules	27.1
